# Fire Performance of Heavyweight Self-Compacting Concrete and Heavyweight High Strength Concrete

**DOI:** 10.3390/ma12050822

**Published:** 2019-03-11

**Authors:** Farhad Aslani, Fatemeh Hamidi, Qilong Ma

**Affiliations:** 1 School of Civil, Environmental, and Mining Engineering, University of Western Australia, Perth, WA 6009, Australia; fatemeh.hamidi.technosa@outlook.com (F.H.); 21847695@student.uwa.edu.au (Q.M.); 2 School of Engineering, Edith Cowan University, Perth, WA 6027, Australia

**Keywords:** fire performance, heavyweight concrete, self-compacting concrete, high strength concrete, heavyweight self-compacting concrete, heavyweight high strength concrete, mechanical properties

## Abstract

In this study, the fresh and hardened state properties of heavyweight self-compacting concrete (HWSCC) and heavyweight high strength concrete (HWHSC) containing heavyweight magnetite aggregate with 50, 75, and 100% replacement ratio, and their performance at elevated temperatures were explored experimentally. For fresh-state properties, the flowability and passing ability of HWSCCs were assessed by using slump flow, T500 mm, and J-ring tests. Hardened-state properties including hardened density, compressive strength, and modulus of elasticity were evaluated after 28 days of mixing. High-temperature tests were also performed to study the mass loss, spalling of HWSCC and HWHSC, and residual mechanical properties at 100, 300, 600 and 900 °C with a heating rate of 5 °C/min. Ultimately, by using the experimental data, rational numerical models were established to predict the compressive strength and modulus of elasticity of HWSCC at elevated temperatures. The results of the flowability and passing ability revealed that the addition of magnetite aggregate would not deteriorate the workability of HWSCCs and they retained their self-compacting characteristics. Based on the hardened densities, only self-compacting concrete (SCC) with 100% magnetite content, and high strength concrete (HSC) with 75 and 100% magnetite aggregate can be considered as HWC. For both the compressive strength and elastic modulus, decreasing trends were observed by introducing magnetite aggregate to SCC and HSC at an ambient temperature. Mass loss and spalling evaluations showed severe crack propagation for SCC without magnetite aggregate while SCCs containing magnetite aggregate preserved up to 900 °C. Nevertheless, the mass loss of SCCs containing 75 and 100% magnetite content were higher than that of SCC without magnetite. Due to the pressure build-up, HSCs with and without magnetite showed explosive spalling at high temperatures. The residual mechanical properties analysis indicated that the highest retention of the compressive strength and modulus of elasticity after exposure to elevated temperatures belonged to HWSCC with 100% magnetite content.

## 1. Introduction

Civil engineering structures are the most expensive investments in a country, therefore, special considerations are needed to preserve their structural integrity and longevity. They may be exposed to various conditions during their service life, which deteriorate their mechanical properties and structural performance. Amongst all, fire is one of the most threatening elements which render safety problems. The compressive strength of normal concrete would fall significantly when exposed to temperatures up to 400 °C [1]. The dramatic decline in the strength caused by elevated temperatures (i.e., above 800 °C) and spalling damage would result in the loss of load bearing capacity of structural elements [2,3], which is mainly due to the chemical decomposition of cement components during exposure to elevated temperatures [4]. Material selection and acquiring knowledge about the high-temperature characteristics of new concrete technology, such as self-consolidating or self-compacting concrete (SCC), high strength concrete (HSC), and heavyweight concrete (HWC), are the fundamentals for minimizing the high-temperature related problems and maintaining the structural integrality and sustainability of concrete structures at fire conditions.

By substituting normal aggregates with denser aggregates, such as barite, hematite, magnetite, iron shot, etc., within cement paste, heavyweight concrete will be produced. In general, the specific gravity threshold for heavyweight aggregates is 3000 kg/m^3^. Concrete with a specific gravity of 2600 kg/m^3^ is categorized as HWC [5]. The electromagnetic shielding effectiveness of HWC makes it an appropriate structural material for nuclear power stations where insulation against radioactive radiation is the foremost concern. However, the sophisticated performance at elevated temperature is of paramount importance in such installations as well. According to Gencel [6], there are two sources of heat generation in nuclear industries, including the internally produced heat owing to the gamma rays and the transferred heat from the hot parts of the reactors. In a research conducted by Horszczaruk et al. [7], the effect of the addition of nanosilica on the mechanical performance of HWC containing quartz, barite, and magnetite aggregates exposed to elevated temperatures were explored. Of these, magnetite-based HWC showed the highest thermal stability, and hence it was concluded that the type of the heavyweight aggregate would determine the thermal resistance of the HWC. Moreover, they revealed that the addition of nanosilica up to 3% would enhance the thermal resistance of the cement mortar containing heavyweight aggregates, especially at temperatures lower than 200 °C [7]. Ling and Poon [8] also revealed that the type of heavyweight aggregate plays a key role in the retention of the mechanical properties of the HWC at elevated temperatures. In barite-based HWC, the thermal conductivity of barite leads to thermal spalling [7] and a dramatic decrease in density and strength of the concrete at 300 °C [8]. In another research, Gencel [6] reported that hematite-based HWC has better compressive strength than plain concrete at elevated temperatures and by increasing the hematite content, reduction in the compressive strength would be lower since the porous and rough surface of hematite aggregate will enhance the bond formation in the interfacial transition zone (ITZ) and therefore improve the mechanical properties of the concrete. Therefore, the type, physiochemical characteristics, and structure of incorporated heavyweight aggregates play important roles in the fire performance of HWC.

SCC is another new-born technology in the concrete industry which has represented outstanding advantages over ordinary concrete since its development in 1986, including better flowability, high resistance to segregation or bleeding, filling capacity, and high strength [9]. For the fabrication of structures with complex shapes or highly congested reinforcement, the superior characteristics of SCC make it an appropriate structural material, since it can flow under its own weight without segregation and easily reach to remote corners [10]. For applications in which high durability is required, HSC is the superior structural material owing to its praiseworthy mechanical properties, including high compressive strength, high stiffness, and high tensile strength [11]. More recently, high-strength SCCs have been the subject of various research [12,13]. A sufficient flowability and resistance to segregation would be provided by means of superplasticizer and viscosity modifying agent while water/binder and fine/coarse aggregate ratios, and maximum aggregate size are other critical influencing parameters [14]. However, despite the growing applications of SCC, HSC, and high-strength SCC, their fire performance and long-term durability as exposed to elevated temperatures still need further research. Spalling and loss of mechanical properties are the major problems associated with high-temperature conditions [15,16,17].

Various researchers reported explosive spalling of SCC in the range of 180–300 °C, leading to the loss of essential mechanical properties [13,18], which is mainly due to the microstructure of SCC. Compared to conventional concrete, SCC contains larger binder content accompanied by chemical components such as silica fume, fly ash, and ground granulated blast furnace slag (GGBFS), which is almost 200 kg/m^3^ more filler than conventional concrete [19]. This will result in a lower permeability and consequently more pressure built-up within SCC increasing the risk of spalling. Pathak and Siddique [20] explored the effect of the addition of class F fly ash on the mechanical properties of SCC at elevated temperatures in the range of 20–300 °C and observed considerable mass loss in the range of 200–300 °C which was accompanied by a dramatic decline in splitting tensile strength due to the departure of bound water. Bakhtiyari et al. [15] studied the fire performance of SCC containing limestone and quartz powder in the range of 150–1000 °C and reported that the temperature range of 480–650 °C is the most critical range for spalling in SCC and normal concrete, and SCC is more susceptible to spalling than normal concrete. However, SCC showed a better retention of mechanical properties at an elevated temperature compared to normal concrete. Li et al. [12] explored the fire performance of HSC at 200, 400, 600, 800, and 1000 °C by means of an oil furnace. They observed a declining trend in the compressive and splitting tensile strengths, and spalling, as yellow, off-white and red straws appeared, starting at 200 °C. Their observations were not in line with other research, especially before 400 °C, due to the different heating profile and the utilization of an electric furnace by other researchers which is not capable of revealing the real situations of fire due to its very low heating rate. Kodur and Sultan [11] pioneered the thermal properties of HSC at elevated temperatures in the range of 0–1000 °C and reported that carbonate aggregates would enhance the fire performance of HSC, and hence concluded, that the type of aggregate can affect the fire performance of HSC significantly.

The main objective of this study is to evaluate the fire performance of magnetite-based heavyweight SCC (HWSCC) and heavyweight HSC (HWHSC), as well as develop numerical models capable of predicting their performance as exposed to elevated temperatures. For that, by substituting magnetite aggregate in the cement paste, two mix series were prepared, of which one represented the characteristics of HWSCC while other one reveled HWHSC characteristics. Thereafter, the prepared mixes were subjected to the fresh- and hardened-state properties to analyze the workability and mechanical properties of HWSCC and HWHSC at the ambient and elevated temperatures including 20, 100, 300, 600, and 900 °C. Ultimately, empirical relationships for compressive strength and modulus of elasticity of HWSCC were proposed and validated by experimental data, which facilitate the assessment of the performance of HWSCC at fire conditions.

## 2. Experiment 

### 2.1. Material

The General Portland (GP) and grade 1 fly ash complying with the requirements of AS3582.1 [21] were used in this experimental study. Ground granulated blast furnace slag (GGFBS) is another supplementary cementitious material used in this study conforming to AS3582.2 [22]. Very fine silica fume was utilized to provide a dense and impermeable concrete in accordance with AS3582.3 [23]. Table 1 summarizes the chemical composition and properties of cement, fly ash, GGFBS, and silica fume. 

Ten mm and <4 mm natural crushed aggregate were used as coarse and fine aggregate, in accordance with AS 1141 (2011), for both SCC and HSC control mixtures. Fine AFS 45-50 silica sand was also used as fine aggregate. Natural magnetite with a density of 3300 kg/m^3^ was utilized with five various sizes including 0.5–1, 1–2, 2–4, 4–6, and 6–10 mm. The former three small particle sizes replaced the <4 mm natural crushed aggregate, as fine aggregate in proportion, whereas the latter two big sizes substituted the <10 mm, as coarse aggregate in the synchronous ratio. All the aggregates utilized in the preparation of concrete mix designs have a diameter of <10 mm, therefore micro-concretes were produced. The properties of natural aggregates and sand are presented in Table 2. The particle distribution of magnetite heavyweight aggregate and its chemical composition are reported in Table 3 and Table 4 respectively.

The superplasticizer admixture (SP) was used that satisfies Type SN chemical admixture. It is designed to improve the flow properties of concrete by lowering the viscosity and yield stress of fresh concrete. High-range water reducer agent (HRWRA) satisfies type HWR. To reduce the difference between disparate content and maintain the homogeneity of the concrete, viscosity modifying admixture (VMA) was added to the mixes which represent SCC characteristics.

### 2.2. Mixing Design

**Figure 1:**
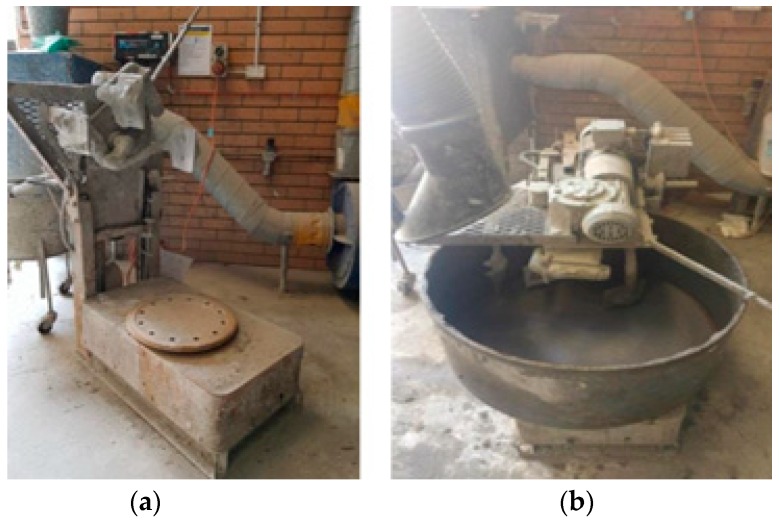
Concrete mixer used for preparation of samples.

Eight concrete mix designs were prepared, in which cement, aggregate, water, and admixtures were the main constituents. A parallel control trial was also adopted. Reducing the water/cementitious materials (w/cm) ratio by means of water-reducing admixtures and superplasticizer, and utilization of high-strength micro-cement containing GGBFS, fly ash, and in particular silica fume, were the main approaches to obtain HSC and SCC. Meanwhile, magnetite heavyweight aggregate was incorporated to produce HWSCC and HWHSC. Figure 1 shows the concrete mixer used in this study.

Based on the absolute volume approach developed by the American Concrete Institute, HWSCC and HWHSC mix designs were prepared [24,25], and then categorized as series 1 and series 2. The specimens in series 1 were designed to comprise SCC with different heavyweight aggregate replacement ratios. This category contained four different proportions of magnetite aggregate including 0, 50, 75, and 100% labeled SCC, HWSCC50, HWSCC75, and HWSCC100 respectively, of which SCC was the control mix design. The mixture proportions of series 1 are shown in Table 5. The prepared specimens in series 2 represented HSC with various heavyweight aggregate replacement ratios, similar to series 1 named HSC, HWHSC50, HWHSC75, and HWHSC100. The mixture proportions of series 2 are shown in Table 6.

The binder compositions of control mixtures of both series 1 and 2 were 51.45% GP cement, 25.7% fly ash, 17.2% GGBFS and 5.7% silica fume. Both control samples were prepared based on 583 kg/m^3^ binder content. The w/cm ratio and binder/aggregate ratio were 0.45 and 0.25 for series 1 and 0.3 and 0.29 for series 2, respectively. 

### 2.3. Fresh-State Properties of SCC

**Figure 2:**
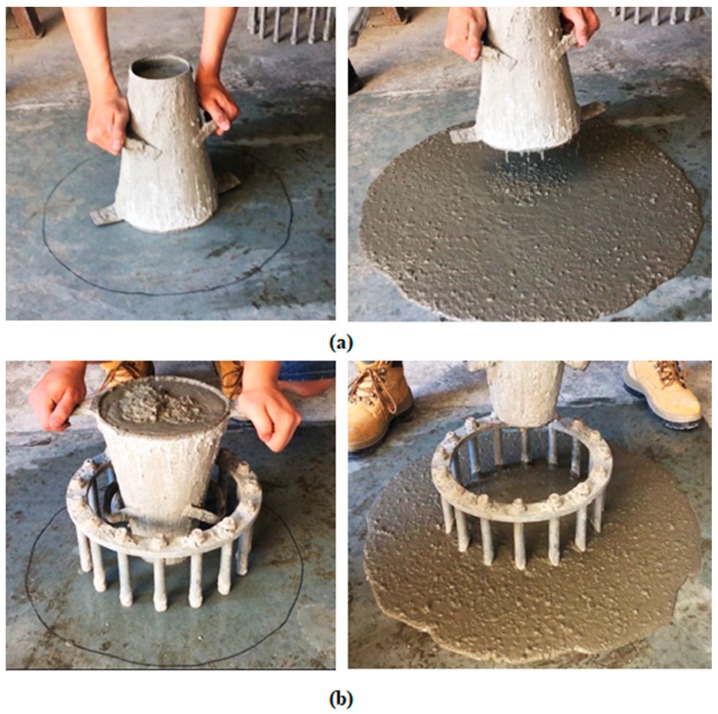
(**a**) Slump test, and (**b**) J-ring test.

The fresh-state characteristics of SCCs (series 1), including the flowability and passing ability were evaluated based on the European Guidelines for SCCs, including the slump test, T_500mm_ time, and J-ring flow test [26]. For HSCs (series 2), only the slump test was conducted due to the negligible difference between the fresh properties of HSC and conventional normal concrete. Figure 2 shows specimen during the slump and J-ring tests.

### 2.4. Hardened-State Properties

To measure the compressive strength and strain gauge test, each mix design required twenty 100 × 200 mm cylinders for five different temperatures (20, 100, 300, 600, and 900 °C) of which, three cylindrical samples were used for the compressive strength test and one sample for the strain gauge test at each temperature. All hardened-property measurements were carried out 28 days after casting. To do this, after demolding after 24 hours, all samples were cured in a curing room with a controlled humidity and temperature for 28 days. For series 1, both the compressive strength and strain gauge test were carried out by a BLH (Baldwin Lima–Hamilton) pressure testing machine. Compared with the compressive strength test, one more vertical strain gauge was attached to the strain gauge test which was parallel to the direction of the applied load. For series 2, the upper force limit of the BLH was 600 kN which was insufficient to reach the breaking stress of HSC. Hence, an ALSMER pressure testing machine was used to complete both tests. Harden densities were evaluated at room temperature while mass loss and spalling were assessed at 100, 300, 600, and 900 °C.

### 2.5. High-Temperature Test

A furnace was used to simulate the fire ignition conditions. For each mixing, four samples were exposed to the fire under each target temperature. The rate of heating was 5 °C/min. Before exposing to high temperatures, the weight of all samples was measured in an ambient temperature (20 °C). After exposure to the target elevated temperatures, the weight of the samples was measured again. The change in weight before and after firing revealed the mass loss of the examined samples. Furthermore, a high temperature leads to a change in the color and occurrence of cracks owing to the weight loss, which are the visual inspections of spalling. To evaluate the residual compressive strength and strain gauge test after heating, all samples must be maintained against explosion after exposure to the target elevated temperatures. These samples were assessed by the same mechanical-property-test procedure at room temperature.

### 2.6. Numerical Modelling

To predict the behavior of concrete at fire conditions, establishing appropriate empirical relationships is essential, since elevated temperatures influence the mechanical properties of the concrete structures, and hence, these impacts should be taken into consideration by engineers to fulfill safety concerns related to the fire performance of concrete structures. More specifically, due to the typical environments where the HWC would be utilized, e.g., nuclear power plants, a deep understanding of the possible changes in the mechanical properties of HWC at elevated temperatures is of paramount importance to maintain the structural integrity of the HWC-based structures. Nevertheless, a limited number of research exploring the relationships for HWC at elevated temperatures is available. Therefore, numerical models for residual compressive strength and moduli of elasticity at high temperature are proposed and validated by experimental results. Moreover, these established relationships are compared with available models built by other researchers to assess their generality and workability for HWC at elevated temperatures.

## 3. Results and Discussion

### 3.1. Fresh-State Properties

**Figure 3:**
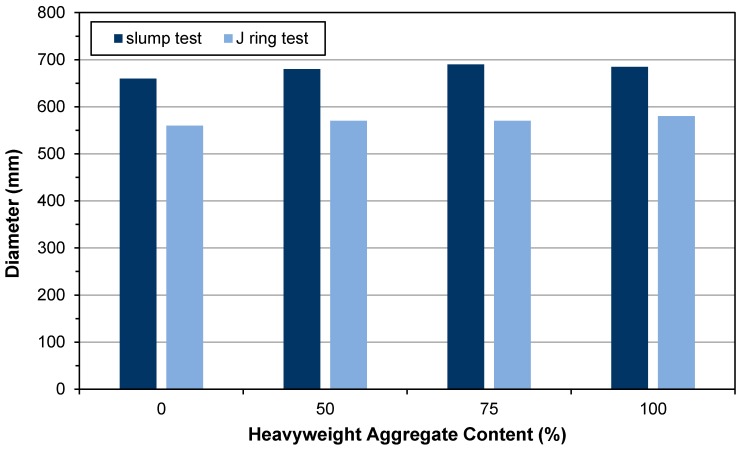
Slump test flow diameter and J-ring flow diameter of series 1.

The obtained results for the slump flow test, which includes the diameter and T_500mm_ time, and J-ring including the diameter and step height (i.e., difference between height of the concrete inside and outside of the J-ring) for HWSCCs are shown in Table 7 and Figure 3. For HWHSCs, the result of the slump flow test is presented in Table 8.

Considering the obtained results from the slump flow test in series 1, all three prepared samples showed a larger spreading diameter than the controlled specimen, of which the largest diameter was 690 mm for HWSCC75. The observed trend suggested that by increasing the heavyweight aggregate content up to 75%, the slump of HWSCC would increase, and afterward, by increasing the magnetite content up to 100% the slump of HWSCC would decrease, as the slump obtained for HWSCC100 is lower than that for the HWSCC75. However, all the examined HWSCC specimens showed a sophisticated slump, since the suggested range of slump was reported to be 600–700 mm for SCC according to the European Guidelines. A slump lower than 500 mm may lead to an insufficient flowability for SCC while a slump value higher than 700 mm would increase the probability of segregation [26,27]. As can be seen in Table 8, in series 2 all the samples containing magnetite aggregate showed a lower spreading diameter than the control sample and by increasing the heavyweight aggregate content, the slump of the HWHSCs decreased. 

For HWC usually low slump values are desired, since low slump values would reduce the bleeding phenomenon in HWC [27,28]. It was reported that increasing the amount of dense magnetite aggregates would result in a decline of the general workability of the mixture [29]. Notwithstanding, increasing the water/binder ratio leads to a promotion in the workability of HWC as revealed by Kilincarslan et al. [30].

The key feature explaining the observed pattern for SCCs is the amount of admixture (i.e., SP, HRWRA, and VMA) incorporated in HWSCC specimens and heavyweight aggregate content. For HWC, the slump value would grow by increasing the admixture dosage, which is evident from HWSCC50 to HWSCC75 specimens. For HWSCC100, the higher magnetite content has led to a lower slump than HWSCC75. The obtained results were in accordance with results reported by Revuelta et al. for barite-based HWSCC [31]. For HSCs, the higher water adsorption of magnetite aggregate than sand resulted in lower slump for HSCs containing 50, 75, and 100% magnetite [5]. 

Results for the T_500mm_, which shows the required time for concrete to reach a diameter of 500 mm after removing the mold, are in complete accordance with the slump values and for all examined specimens are less than 3 s. Higher slump values resulted in lower T_500mm_ for HWSCC50 and HWSCC75. HWSCC100 had the highest T_500mm_ time which may be attributed to the high amount of heavyweight magnetite aggregates. 

Considering the obtained results from the J-ring test, for all three HWSCC the obtained diameters are higher than that of control SCC sample, and all of them are in the range of 550–600 mm, which is described as the appropriate range for SCC by the European Guidelines. Moreover, the step height results revealed that by increasing the heavyweight magnetite content, the step height reduced, since the HWSCC100 had the lowest step height equal to 13 mm, while the control sample had the highest step height equal to 17 mm. This result showed that by increasing the VMA dosage, the homogeneity of the HWSCC would increase [32]. 

Therefore, it can be claimed that the substitution of normal aggregates by heavyweight magnetite aggregates would not negatively affect the flowability and passing ability of SCC since the slump flow and J-ring tests showed a sufficient workability for the prepared specimens to be considered as HWSCC.

### 3.2. Hardened-State Density

**Figure 4:**
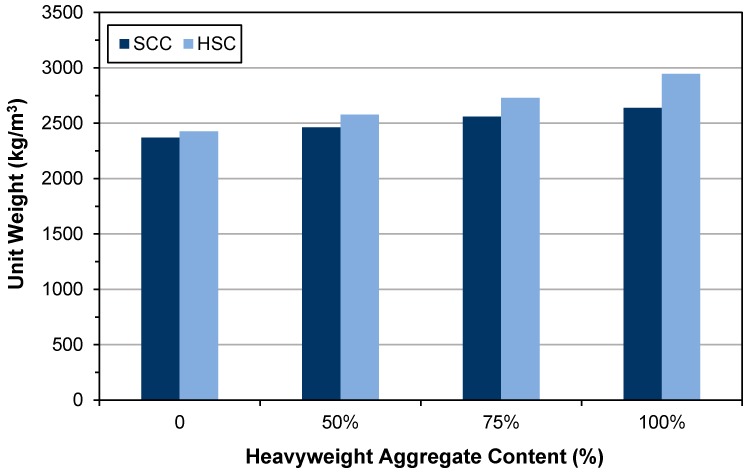
Hardened-state densities of series 1 and 2.

The hardened-state densities of both series 1 and 2 are given in Table 9 and shown in Figure 4. Similar to previous research conducted by Kilincarslan et al. [30], the obtained results in this study revealed that by increasing the heavyweight aggregate content in both series 1 and 2, the dry density of the mixture would increase. As mentioned by various researchers, the appropriate range of density for HWC is 2600–6000 kg/m^3^ [29,33]. Therefore, only HWSCC100, and HWHSC with 75% and 100% magnetite substitution can be considered as HWC. 

### 3.3. Spalling Observation

Spalling is the removal of pieces of concrete from the surface of a structure [34,35]. To report spalling observation, the surface condition of the samples, including cracks and color changes, were recorded, and the observed results are presented in Table 10 and Table 11.

As can be seen, no visible changes were observed for both series 1 and 2 up to 300 °C. For series 1, SCC showed severe crack propagation and color changing at 600 °C, while other samples, containing 50, 75, and 100% heavyweight magnetite content, remained well-preserved up to 900 °C. In the study conducted by Persson [19], explosive spalling was observed for all the SCC specimens without polypropylene fiber. Other researchers also reported explosive spalling for SCC in the range of 180–250 °C, even at a low heating rate of 0.5 °C/min [13]. Moreover, as is evident from Table 10, there was no obvious cracking during heating up to 600 °C for HWSCC50, HWSCC75, and HWSCC100. For these three samples, visible spalling and color change occurred at 900 °C, which resulted in brittleness of the cylinders.

As Hertz [2] explained, a dense reinforcement and aggregate, high heating rate, thermal stresses and thermal gradient accompanied with moisture gradient, low permeability, and closed pores containing water within cement matrix are the most probable factors that contributed to the explosive spalling of concrete structures. The thermal expansion of heavyweight aggregates renders a mismatch between the aggregates and surrounding cement matrix and the breakdown of interfacial bonds in the vicinity of ITZ, and therefore, a significant propagation of micro-cracks within the concrete structure [36]. High binder content and the addition of silica fume can also increase the risk of explosive spalling, since even at low water/cement (w/c) ratio, high cement content would require more water whereas addition of silica fume leads to a decline in the permeability of the cement matrix [35]. This could be well described the extreme spalling observation for SCC sample. 

Compared to barite aggregate, magnetite has lower thermal conductivity, however, it still has a higher thermal conductivity than normal aggregate. As reported by the authors of Reference [37], the thermal conductivity of magnetite aggregate is in the range of 6–23 W/m.K, while for normal aggregate it is in the range of 1.3–2.8 W/m.K at ambient temperature which would explain the minor crack propagation for HWSCCs at higher temperatures (i.e., 900 °C) compared to SCC. Higher thermal conductivity results in a better heat transfer, and consequently will reduce thermal stresses within the concrete, which are originated from the temperature gradients between the inner and outer surfaces of the concrete structure exposing to high temperature. Hence, magnetite aggregate with higher thermal conductivity than normal aggregate, would lessen the risk of explosive spalling significantly. This reveals the reason for only observing micro-cracks for SCCs containing magnetite aggregate even at 900 °C without extreme spalling. 

For series 2, all samples of the control HSC and HWHSC containing 50% magnetite content were exploded at 600 °C, as shown in Table 11. The obtained results are in accordance with Kodur’s researches [11,38]. Spalling would occur due to the pore pressure build-up, thermal stresses, and a combination of them during heating [33]. HSC is more prone to pressure build-up owing to its low permeability compared to normal strength concrete [35,38]. When temperatures exceed 300 °C, such an internal pressure within HSC grows rapidly [39]. Even by substituting a normal aggregate with heavyweight magnetite aggregate, explosive spalling occurred, as can be seen for HWHSC50 [38]. Hence, the HWHSC75 and HWHSC100 have been no longer considered for high temperature tests. 

### 3.4. Mass Loss

**Figure 5:**
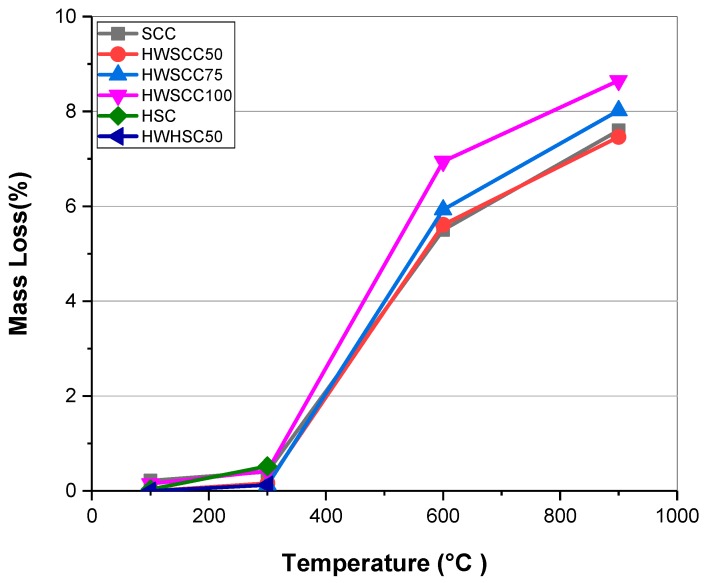
Average mass loss of self-compacting concrete and high strength concrete with different heavyweight aggregate replacement ratio mixes at elevated temperatures.

Mass loss was obtained from the difference between the weight before and after heating. At elevated temperatures, the mass loss occurred mainly due to the dehydration, thermal decomposition of the cement components, and spalling from the surface layer [4,40]. It develops crack propagation, and can even result in explosion. Figure 5 represents the mass loss of examined samples at elevated temperatures.

At 100 °C and 300 °C no significant mass loss occurred for examined specimens, however, the evaporation of the bound water is the main reason for the observed low mass loss percentage in this temperature range [6,41]. Nevertheless, a dramatic increase in mass loss is evident at 600 °C for SCCs without and with 50%, 75%, and 100% magnetite content. Their mass loss were more than 10 times compared to 300 °C and reached 5.5%, 5.6%, 5.9%, and 6.9% respectively. By increasing the temperature up to 900 °C, the mass loss of SCC, HWSCC50, HWSCC75, and HWSCC100 reached 7.6%, 7.5%, 8%, and 8.6% respectively. Regarding HSC samples, the HWHSC50 showed lower mass loss than the control HSC sample at 300 °C. Therefore, the fire resistance ability would be promoted by replacing half of normal aggregates with heavyweight aggregates in both SCC and HSC. However, increasing the amount of heavyweight aggregates would result in more mass loss for the HWSCC, since the specimen with 100% magnetite content showed the largest mass loss percentage amongst all the examined samples at high temperatures. According to Reference [28], at a high binder content and high w/c ratio (i.e., more than 0.4), increasing the VMA content would increase the relative water absorption of the magnetite-based HWC. The VMA content has been increased by increasing the magnetite content in the prepared HWSCCs in this experiment. Higher water absorption would result in higher water release, and subsequently higher mass loss for specimens with higher magnetite content.

According to the previous research conducted by Horszczaruk et al. [7], HWC containing magnetite aggregate showed lower mass loss than those containing quartz and barite aggregates. In their experiment, mass loss of magnetite-based concrete was almost twice as low as quartz-based HWC, which was attributed to the better thermal stability of magnetite aggregates at high temperatures [42]. Ling et al. [8] reported s high mass loss for barite-based HWC in the temperature range up to 300 °C owing to the high water absorption capacity and thermal conductivity of barite aggregates. In another research conducted by Gencel [6], hematite aggregate was substituted in the cement paste and the obtained results revealed that the mass loss of hematite-based cement was lower than that of plain concrete at elevated temperature contributed to the elemental composition of hematite which is rich in iron. Therefore, the thermal stability, composition, and relative water absorption of the heavyweight aggregate are the governing factors determining the mass loss of HWC.

### 3.5. Initial and Residual Compressive Strength

**Figure 6:**
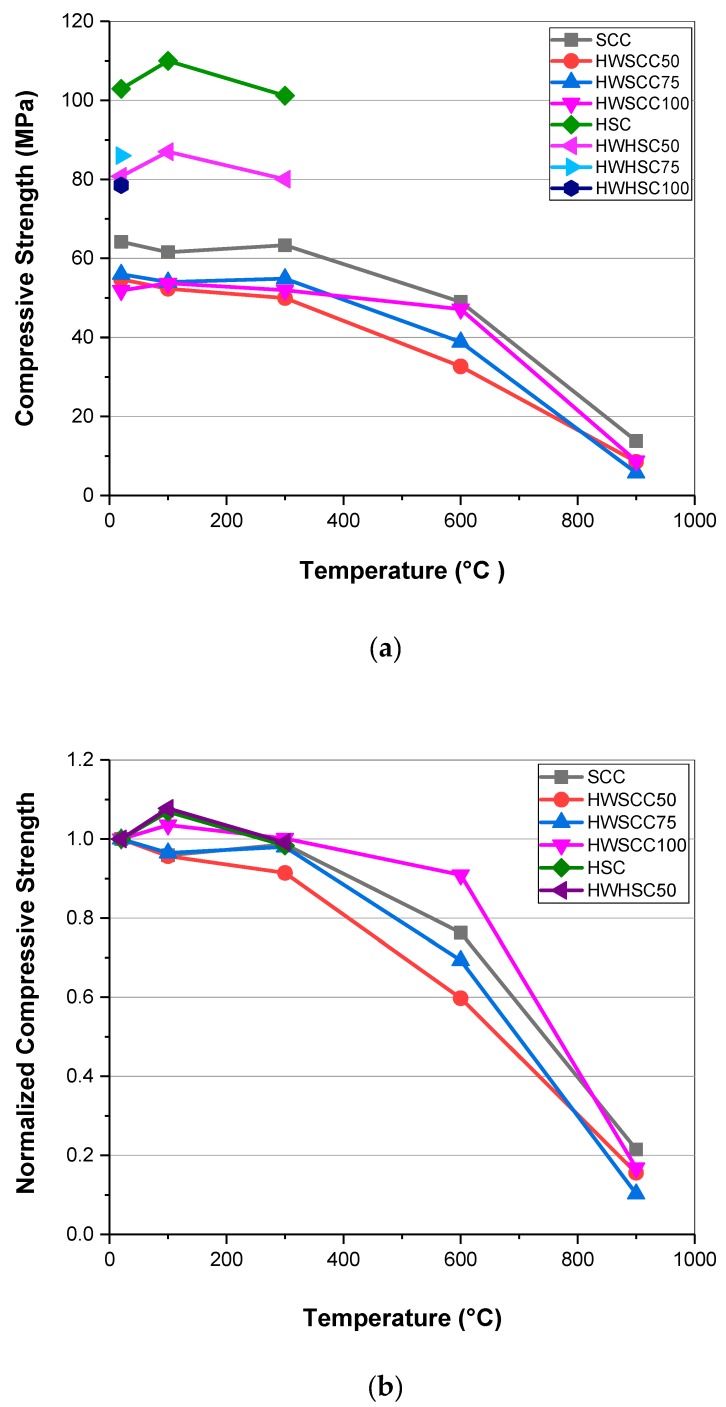
(**a**) Initial and 28-day residual compressive strength at elevated temperatures (**b**) normalized value of compressive strength.

The initial and residual compressive strength of SCCs and HSCs after 28 days are shown in Figure 6. The 28-day compressive strength results for series 1 at ambient temperature range from 64.2 MPa, for 0% magnetite content, to 51.9 MPa for 100% magnetite replacement. Compared to 0% magnetite content, substituting 50, 75, and 100% heavyweight aggregate declined the compressive strength of the specimens by 14.8, 12.8, and 19.2%, respectively.

Similarly, the 28-day compressive strength of specimens in series 2 dropped by increasing the magnetite content. The specimen with 0% heavyweight aggregate showed a compressive strength of almost 103 MPa which satisfies the 60 MPa threshold for HSC [43,44]. By replacing half of the coarse aggregates with magnetite, the compressive strength was reduced by one fifth to 80 MPa. Afterwards, by increasing the magnetite content up to 75 and 100%, small fluctuations in compressive strength were observed. However, all the mixture designs showed compressive strength of more than 60 MPa.

At high w/c ratio, for instance more than 0.4, there was no significant difference between the compressive strength of HWSCC with SCC. However, by increasing the heavyweight aggregate the compressive strength of HWSCC decreased more than SCC due to the structure of magnetite aggregate which contained weak planes unable to withstand the compressive failure. At low w/c ratio (i.e., less than 0.4), as it can be seen in the series 2, there was a significant difference in the compressive strength of HSC to that of HWHSC, since at low w/c the internal structure of the cement matrix without heavyweight aggregates would improve, and therefore, the heavyweight magnetite aggregates have a negative impact on the compressive strength. Similar findings have been reported by Mostofinejad et al. [45] in which they observed lower compressive strength for barite-based HWC than concrete with normal limestone aggregates at lower w/c ratio but no significant difference at higher w/c ratio. These results are further confirmed by Reference [46].

Figure 6b represents the normalized compressive strength based on the compressive strength of each specimen at ambient temperature. Considering the residual compressive strength at elevated temperature, HSCs with 75 and 100% magnetite have not been evaluated since they exploded and HSC and HWHSC50 exploded by increasing temperature more than 300 °C. Therefore, in series 2 only HSC and HWHSC50 were subjected to the compressive strength assessment up to 300 °C. For SCCs before 300 °C there was no obvious change in the compressive strengths of all mixes. By raising the temperature from 300 to 600 °C, a declining trend was observed, particularly for the control SCC sample. As temperature hit 900 °C, a dramatic decline in the compressive strength of all specimens occurred. Even a sharp decrease for HWSCC100 was observed and its compressive strength reduced to 8.7 MPa, meaning that it only retained 18.5 and 16.8% of its strength in 600 °C and 20 °C, respectively. For HSCs, an initial increase in the compressive strength of both samples was observed at around 100 °C followed by a decrease at 300 °C. Horszczaruk et al. [42] reported similar results for magnetite-based concrete at elevated temperatures in which the specimen containing 100% magnetite aggregate retained the highest compressive strength at elevated temperatures representing 79% and 40% its initial strength at 600 and 800 °C, respectively. Up to 300 °C, the further hydration of unhydrated cement particles due to an internal autoclaving effect caused by high temperature and evaporation of water [47,48] prevented significant changes in the compressive strength of SCC specimens, and a growth in the compressive strength of HSCs [49]. Probably the low permeability and low air content in HSCs led to better heat transfer compared to SCCs, since the conductivity of concrete is almost 70 times higher than air [50], and thus further hydration and propagation of C-S-H gel, and consequently an initial improvement in the compressive strength of HSCs occurred. By increasing the temperature, the compressive strength of SCC and HSC declined due to the spalling effect [7] while for HWSCCs and HWHSC50, thermal expansion of magnetite aggregates results in better retention of compressive strength compared to SCC and HSC at the same temperature, as can be seen in Figure 6b [2]. However, at 600 and 900 °C, this thermal expansion lead to the significant destruction of bonds between magnetite aggregates and cement paste in their ITZ, and hence the loss of compressive strength for HWSCCs and HWHSC50 in comparison with the ambient temperature [42]. Moreover, the thermal decomposition of cement constituents exacerbated the deteriorating trend in the mechanical properties of all samples at 600 and 900 °C. The rehydration of lime was accompanied by a significant expansion, and therefore a dramatic decline in the compressive strength above 350 °C. The decomposition of C-S-H gel also started at around 560 °C which resulted in a slight reduction in the compressive strength [4,48,51].

### 3.6. Initial and Residual Modulus of Elasticity

**Figure 7:**
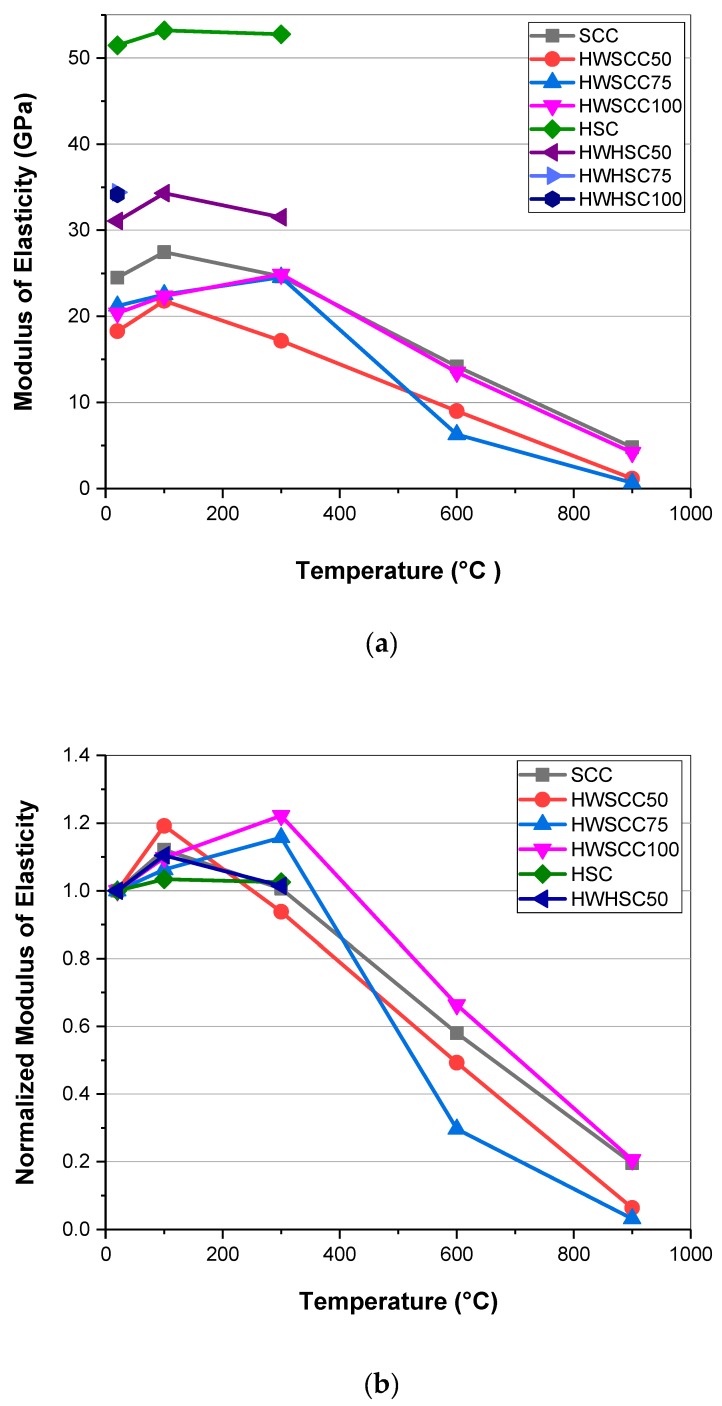
(**a**) Initial and 28-day residual modulus of elasticity at elevated temperatures, (**b**) normalized value of modulus of elasticity.

The initial and residual modulus of elasticity of SCCs and HSCs after 28 days are shown in Figure 7. The elastic modulus of SCC has a clear drop when magnetite aggregates were incorporated. The lowest deformation resistance appeared for HWSCC50, which is a loss of 25.3% of its modulus of elasticity compared to the control mix. By increasing the magnetite content to 75 and 100%, the modulus of elasticity increased slightly. A similar trend was evident for the HSCs. Nevertheless, the decline of the elastic modulus for HSC by incorporating heavyweight magnetite aggregates was more significant since two fifths of modulus was lost by substituting 50% magnetite in HSC. However, since the HSC had a high modulus of elasticity, even the lowest elastic modulus belonging to HWHSC50 was 31.057 GPa.

In general, by replacing normal aggregates with heavyweight aggregates, the ability of the cement matrix to resist deformation is reduced owing to the highly crystalline microstructure of the heavyweight aggregates containing weak planes [46]. Moreover, the generation of voids and therefore, the formation of a porous ITZ around the aggregates leads to a reduction in both the compressive strength and elastic modulus of mixtures containing heavyweight aggregates [52]. Similar to the results obtained for the compressive strength, probably due to the lower w/c ratio applied for HWHSCs, the difference between the control sample and HWHSC samples with different magnetite contents are more significant than that observed for HWSCCs with a higher w/c ratio.

Figure 7b showed the normalized modulus of elasticity of SCCs and HSCs based on the modulus of elasticity of each specimen at ambient temperature. Regarding residual modulus of elasticity for SCCs, unlike the observed pattern for the compressive strength, modulus of elasticity of all specimens experienced an increase during relatively low temperature situation, and then the trend reversed thereafter. For control SCC sample and HWSCC50, the turning point was occurred at 100 °C, while for HWSCC75 and HWSCC100, the turning point was pushed to 300 °C, after which all samples showed a drop. After reaching 900 °C, HWSCC100, which had represented the highest modulus of elasticity amongst all, only retained one fifth of its initial stiffness. Similar to SCCs, HSC and HWHSC50 showed an increase in the modulus of elasticity from 20 to 100 °C, followed by a decrease from 100 to 300 °C. 

Rapid drying accompanied by a further hydration of unhydrated cement particles, and hence the improvement of mechanical properties of C-S-H gel [7,51], result in an initial increase in the elastic modulus of the samples. For both HWSCCs and HWHSC50, the initial increase of the modulus of elasticity are more significant than for SCC and HSC, as can be seen in Figure 7b. Moreover, for HWSCC75 and HWSCC100 the initial increase in the elastic modulus occurred at a higher temperature compared to the control SCC sample and HWSCC50. A higher thermal conductivity of magnetite aggregates than cement and air in the filling of pores within the cement matrix can well explain the more significant initial increase of modulus of elasticity for HWSCCs and HWHSC50 [37]. Increasing the magnetite content, especially with a smaller particle size, leads to the filling of pores and a more compact microstructure. Pore compaction and higher thermal conductivity of magnetite aggregate result in a better heat transfer, and subsequently the further hydration of unhydrated cement particles than those samples without magnetite aggregates. The higher amount of absorbed water [28] and thermal stability of magnetite aggregates [8] caused the further hydration, and therefore the initial growth in the modulus of elasticity for HWSCC75 and HWSCC100 occurred at a higher temperature. Similar to the compressive strength, by increasing the temperature, spalling and micro-crack propagation within the cement structure and at the ITZ between cement matrix and aggregates occurred due to the pressure build-up, shrinkage [52,53], and the thermal expansion of magnetite aggregates. After reaching 400 °C, micro-cracks within the cement matrix propagated noticeably, accompanied by the dehydration of calcium hydroxide to calcium oxide, which resulted in a more than 44%-decrease in volume, and subsequently a dramatic decline in the elastic modulus [7,42]. 

## 4. Numerical Modelling

Developing numerical models to describe stress-strain curves for concrete at elevated temperatures is of great importance [54,55,56,57,58,59,60,61,62] since, from one hand, it would not be an easy-achieving task to measure the mechanical properties in such high-temperature range, particularly above 300 °C, and from the other hand, HWC-based structures usually will expose to high temperatures due to the places where they would be installed. Herein, based on the experimental data and regression analysis, empirical relationships for residual compressive strength and modulus of elasticity of HWSCC containing magnetite aggregate at elevated temperatures have been proposed in the Equations (1) and (2). According to the results obtained for hardened-state density, only SCC containing 100% magnetite content can be considered as HWSCC, therefore, the regression analyses were conducted based on the experimental results for HWSCC10.

(1) $$f_{CT}^{\prime }=f_{C}^{\prime }\left(-3.062\times 10^{-9}T^{3}+2.1085\times 10^{-6}T^{2}-3.66\times 10^{-4}T+1.02367\right)\;20\;℃\leq T\leq 900\;℃$$

(2) $$E_{CT}^{\prime }=E_{C}^{\prime }\left(1.322\times 10^{-11}T^{4}-1.83\times 10^{-8}T^{3}+3.2\times 10^{-6}T^{2}+9.747\times 10^{-4}T+0.97\right)\;20\;℃\leq T<600\;℃\;E_{CT}^{\prime }=E_{C}^{\prime }\left(-1.53\times 10^{-3}T+1.5785\right)\;600\;℃\leq T\leq 900\;℃$$

In which $f_{cT}^{\prime }$ and $E_{CT}^{\prime }$ are the residual compressive strength and modulus of elasticity, and $f_{c}^{\prime }$ and $E_{C}^{\prime }$ are the compressive strength and modulus of elasticity at ambient temperature, respectively.

**Figure 8:**
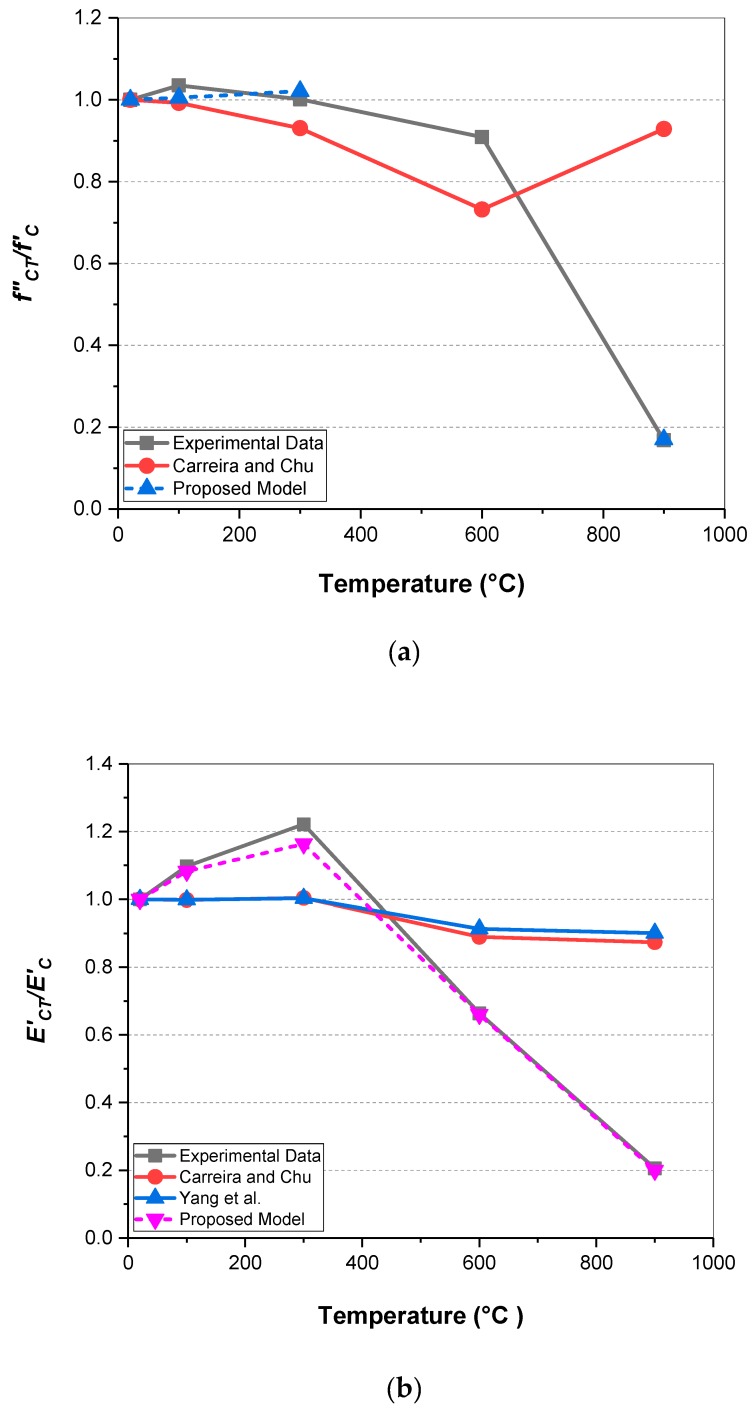
(**a**) comparison between the proposed model for compressive strength of HWSCC with available equations and experimental data, (**b**) comparison between the proposed model for modulus of elasticity of HWSCC with available equations and experimental data.

Table 12 represents available relationships for compressive strength and modulus of elasticity of unconfined concrete in compression at ambient temperature which are applicable for HWC with density up to 4500 kg/m^3^ [63]. Figure 8 shows the obtained results from these relationships compared with the obtained results from the proposed relationships. 

The only available relationship which can be used to predict the compressive strength of HWSCC containing magnetite at elevated temperature was stablished by Carreira and Chu [64]. However, this model predicts the compressive strength as a function of strain. As can be seen in Figure 8a, the proposed model by Carreira and Chu [64] is only capable of predicting the decreasing trend in the compressive strength up to 600 °C, and it is not applicable for temperatures above 600 °C. 

Regarding the modulus of elasticity, Carreira and Chu [64] and Yang et al. [63] models are based on the density of the specimen, since the modulus of elasticity increases by increasing the density, especially for values higher than 2500 kg/m^3^ [63]. However, as can be seen in Figure 8b, these models are not able to predict the behavior of HWSCC at elevated temperatures since the $E_{CT}^{\prime }$ soared by losing mass, and subsequently density of HWSCC containing 100 magnetite aggregate up to 300 °C is reduced. Above 300 °C, both density and $E_{CT}^{\prime }$ plunged but since the change in the mass at elevated temperature is not as significant as the observed declining trend in the modulus of elasticity, hence the available models cannot predict the modulus of elasticity for HWSCC at elevated temperatures accurately. The proposed model, which only considered the change in the modulus of elasticity by increasing the temperature, reveals a better workability than the reference models. Nevertheless, considering both the density and effect of temperature on $E_{CT}^{\prime }$ results in a more accurate model for the modulus of elasticity of HWSCC at elevated temperatures, which needs further research.

## 5. Conclusions

This experimental study evaluates the performance of magnetite-based HWSCC and HWHSC at elevated temperatures ranging from 100–900 °C. Based on the obtained results, the following conclusions may be drawn:

1. Heavyweight magnetite aggregate will not deteriorate the self-compacting characteristics of SCC containing magnetite since the HWSCCs with different proportions of magnetite showed sufficient flowability and passing ability.
2. The introduction of magnetite aggregates reduces the compressive strength and modulus of elasticity for both SCC and HSC due to the structure of magnetite aggregate and formation of voids around aggregates in the vicinity of the ITZ. At a lower w/c ratio (lower than 0.4), the declining trend is more significant since at low w/c ratio the internal structure of the cement paste without heavyweight aggregate will improve.
3. At 300 °C, no significant spalling was observed for HWSCC and HWHSC. By reaching 600 °C, SCC, HSC, and HSC containing 50% magnetite spalled explosively due to the high cement content, high heating rate and pressure build-up owing to their low permeability. HWSCCs with 50%, 75%, and 100% magnetite withstood high temperature up to 900 °C. However, at 900 °C, cracks occurred on the surface of the samples and they became brittle owing to the thermal expansion of heavyweight aggregates promoting crack-propagation within the cement matrix.
4. Through the evaporation of bound water in the range of 100–300 °C, low mass loss occurred for all the examined samples. At 600 °C, a dramatic increase in the mass loss is evident for SCC containing 50%, 75%, and 100% magnetite, and by increasing the magnetite content, more mass lose would occur. The observed trend was probably contributed to the amount of VMA added to the HWSCC samples. At high w/c ratio and high binder content, by increasing the VMA dosage, the relative water absorption will increase, therefore, more mass loss would occurred for SCC with higher magnetite content at elevated temperatures.
5. Due to the shrinkage, the thermal decomposition of cement components, spalling, and the disruption of bonds at ITZ between the aggregate and cement matrix, a dramatic decrease in the mechanical properties of SCCs without and with magnetite aggregate occurred at 600 and 900 °C. Nevertheless, by increasing the magnetite content, the residual compressive strength and modulus of elasticity for SCCs containing magnetite aggregate at elevated temperatures was higher, which was probably due to the thermal expansion of magnetite aggregates.
6. The compressive strength–temperature relationship of HWSCC is probably in the form of a cubic equation while the modulus of elasticity can be expressed as a function of temperature by a fourth-order equation in the range of 20–600 °C and a linear equation in the range of 600–900 °C. However, more investigation is required to determine the impact of heavyweight aggregate content and its density on these relationships.

## Figures and Tables

**Table 1:** Properties of cement, fly ash, slag, and silica fume.

**General Purpose Cement**		**Fly Ash**	
**Chemical Properties**	**Value**	**Chemical Properties**	**Value**
CaO	63.40%	CaO	3.30%
SiO_2_	20.10%	SiO_2_	50.40%
Al_2_O_3_	4.60%	Al_2_O_3_	31.50%
Fe_2_O_3_	2.80%	Fe_2_O_3_	10.40%
SO_3_	2.70%	SO_3_	0.10%
MgO	1.30%	MgO	1.10%
Na_2_O	0.60%	Na_2_O	0.30%
Total Chloride	0.02%	K_2_O	0.50%
**Physical Properties**	**Value**	SrO	<0.1%
Specific Gravity	3.0–3.2 t/m^3^	TiO_2_	1.90%
Fineness index	390 m^2^/kg	P_2_O_5_	0.50%
Normal consistency	27%	Mn_2_O_3_	0.20%
Setting time initial	120 min	Total Alkali	0.60%
Setting time final	210 min	**Physical Properties**	**Value**
Soundness	2 mm	Relative Density	2.29
loss on ignition	3.80%	Moisture	<0.1%
Residue 45μm sieve	4.70%	Loss on Ignition	1.10%
**Mechanical Properties**	**Value**	Sulphuric Anhydride	0.10%
Mortar Comp Str.	-	Chloride Ion	0.00%
*f′_c_* 3 Days	38.6 MPa	Chemical Composition	92.30%
*f′_c_* 7 Days	48.4 MPa	Relative Water Requirement	93%
*f′_c_* 28 Days	58.5 MPa	Strength index	102%
Shrinkage 28 days	640 μ strain	-	-
**Ground Granulated Blast Furnace Slag**		**Silica Fume**	
**Chemical Properties**	**Value**	**Chemical Properties**	**Value**
S	0.40%	Silicon as SiO_2_	98%
SO_3_	2.40%	Sodium as Na_2_O	0.33%
MgO	5.70%	Potassium as K_2_O	0.17%
Al_2_O_3_	12.60%	Available Alkali	0.40%
FeO	0.80%	Chloride as Cl^-^	0.15%
MnO	0.10%	Sulphuric Anhydride	0.83%
Cl	0.01%	Sulphate as SO_3_	0.90%
Insoluble residue content	0.20%	**Physical Properties**	**Value**
**Physical Properties**	**Value**	Bulk Density	625 kg/m^3^
Specific Gravity	3.0–3.2	Relative Density	2.21
Relative Water requirement	103%	Pozzolanic Activity at 7days	111%
Relative Strength	100%	Control Mix Strength	31.3 MPa
Temperature rise	18.8 °C	Moisture Content	1.10%
Fineness (passing 45 μm)	98%	Loss of Ignition	2.40%

**Table 2:** Properties of natural magnetite aggregate and sand.

**Natural Crushed Aggregate 10 mm**		**Natural Crushed Aggregate < 4 mm**	
**Sieve Size**	**% passing**	**Sieve Size**	**% passing**
13.2 mm	100%	4.75 mm	100%
9.5 mm	87%	2.36 mm	80%
6.7 mm	20%	1.18 mm	55%
4.75 mm	7%	600 μm	39%
2.35 mm	4%	300 μm	27%
1.18 mm	3%	150 μm	18%
600 μm	2%	75 μm	13%
300 μm	2%	-	-
150 μm	2%	Apparent Particle Density	2.76 t/m^3^
75 μm	2%	Particle Density Dry	2.65 t/m^3^
-	-	Particle Density SSD	2.69 t/m^3^
Moisture Content	0.5%	Water Absorption	1.40%
Flakiness Index	24.0%	Moisture Content	2.50%
**AFS 45-50 Silica Sand**		**AFS 45-50 Silica Sand**	
**Chemical Constituents**	**Value**	**Sieve Size**	**% Retained**
SiO_2_	99.86%	850μm	0%
Fe_2_O_3_	0.01%	600μm	0.30%
Al_2_O_3_	0.02%	425μm	11.90%
Cao	0.00%	300μm	40.80%
MgO	0.00%	212μm	31.60%
Na_2_O	0.00%	150μm	12.60%
K_2_O	0.00%	106μm	2.30%
TiO_2_	0.03%	75μm	0.20%
MnO	<0.001%	-	-
Loss on Ignition	0.01%	-	-
Water Content (@105 °C)	<0.001%	-	-
AFS Number	47.50%	-	-

**Table 3:** Particle distribution of magnetite aggregate.

Characteristic Sieve Size	Percentage Passing (%)					
	AFS 45/50 Silica Sand	Heavyweight Aggregates (Magnetite)				
		(0.5–1) mm	(1–2) mm	(2–4) mm	(4–6) mm	(6–10) mm
13.2 mm	-	-	-	-	100.0	60.0
9.5 mm	-	-	-	-	100.0	14.7
6.7 mm	-	-	-	-	70.3	0.0
4.75 mm	-	-	100.0	100.0	15.7	-
2.36 mm	-	100.0	98.0	95.8	0.0	-
1.18 mm	100.0	98.0	14.7	8.5	-	-
0.6 mm	99.0	14.7	3.9	0.0	-	-
0.3 mm	23.8	3.9	2.0	-	-	-
0.15 mm	1.3	2.0	1.0	-	-	-
0.75 mm	0.0	-	0.0	-	-	-

**Table 4:** Chemical composition of magnetite aggregate.

Properties	Value
**Chemical**	
Fe	>95.5%
Si	2.20%
C	0.50%
Mn	2.20%
**Physical**	
Hardness	5.1
Specific Gravity	4.6 g/cm^3^

**Table 5:** Self-compacting concrete with heavyweight aggregate replacement mix proportions (series 1).

SCC: Heavyweight Aggregates Mix Proportion	Control SCC	HWSCC50	HWSCC75	HWSCC100
**Binder (kg/m^3^)**				
GP Cement	300	300	300	300
Fly Ash	150	150	150	150
GGBFS	100	100	100	100
Silica Fume	33	33	33	33
Cementitious materials	583	583	583	583
Water	262.35	262.35	262.35	262.35
**Aggregates (kg/m^3^)**				
Fine silica sand AS 45-50	360	360	360	360
<4 mm normal	1050	525	262.5	350
<10 mm normal	900	450	225	350
0.5–1 mm magnetite	-	173	262.5	350
1–2 mm magnetite	-	173	262.5	450
2–4 mm magnetite	-	173	262.5	450
4–6 mm magnetite	-	225	337.5	-
6–10 mm magnetite	-	225	337.5	-
**Admixtures (L/m^3^)**				
Superplasticizer	6	2.5	2.875	2.75
High-range water reducer	1.9	0.75	0.75	0.875
Viscosity modifying agent	0.6	-	0.75	1.875

**Table 6:** High strength concrete with heavyweight aggregate replacement mix proportions (series 2).

HSC: Heavyweight Aggregates Mix Proportion	Control HSC	HWHSC50	HWHSC75	HWHSC100
**Binder (kg/m^3^)**				
GP Cement	300	300	300	300
Fly Ash	150	150	150	150
GGBFS	100	100	100	100
Silica Fume	33	33	33	33
Cementitious materials	583	583	583	583
Water	175	175	175	175
**Aggregates (kg/m^3^)**				
Fine silica sand AS 45-50	260	260	260	260
<4 mm normal	950	475	237.5	-
<10 mm normal	800	400	200	-
0.5–1 mm magnetite	-	158.5	237.5	317
1–2 mm magnetite	-	158.5	237.5	317
2–4 mm magnetite	-	158.5	237.5	317
4–6 mm magnetite	-	200	300	400
6–10 mm magnetite	-	200	300	400
**Admixtures (L/m^3^)**				
Superplasticizer	7.5	7.5	6.25	5
High-range water reducer	1.25	1.25	0.75	0.75

**Table 7:** Fresh property test results for series 1.

Mix	SCC	HWSCC50	HWSCC75	HWSCC100
**Slump flow** **diameter (mm)**	665	685	690	680
**T_500mm_ time (s)**	2.13	2.01	2.06	2.31
**J-ring diameter (mm)**	570	575	580	585
**J-ring height difference (mm)**	17	15	14	13

**Table 8:** Slump flow result for series 2.

Mix	HSC	HWHSC50	HWHSC75	HWHSC100
**Slump flow diameter (mm)**	243	236	225	217

**Table 9:** Hardened-state densities of series 1 and 2.

**Series 1. SCC category**				
**Mix**	**SCC**	**HWSCC50**	**HWSCC75**	**HWSCC100**
**Mass (kg)**	3.724	3.869	4.02	4.14
**Density (kg/m^3^)**	2370.6	2462.9	2559.2	2638
**Series 2. HSC category**				
**Mix**	**HSC**	**HWHSC50**	**HWHSC75**	**HWHSC100**
**Mass (kg)**	3.813	4.05	4.29	4.62
**Density (kg/m^3^)**	2427.4	2578.3	2729.2	2945.6

**Table 10:** Spalling observation results for series 1 (self-compacting concrete).

MIX	100 °C	300 °C	600 °C	900 °C
SCC	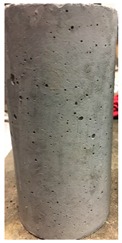	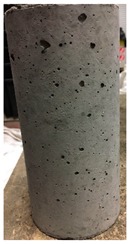	Sever crack propagation and color changing	Sever crack propagation and color changing
HWSCC50	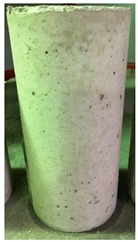	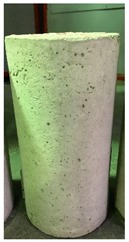	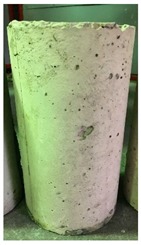	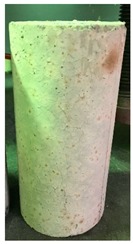
HWSCC75	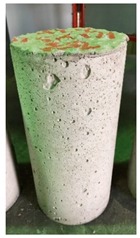	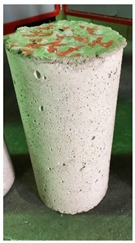	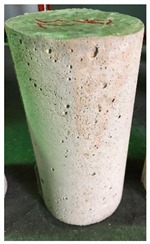	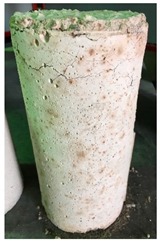
HWSCC100	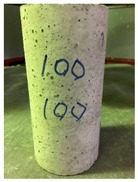	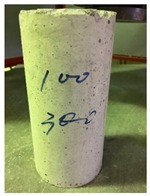	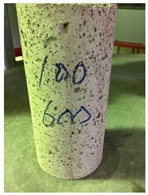	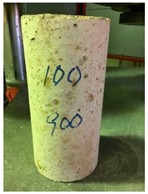

**Table 11:** Spalling observation results for series 2 (high strength concrete).

MIX	100 °C	300 °C	600 °C	900 °C
HSC	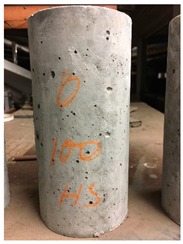	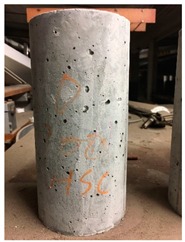	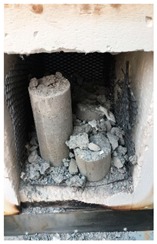	Exploded
HWHSC50	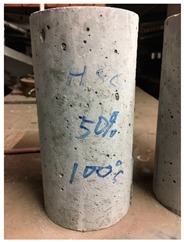	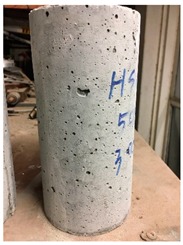	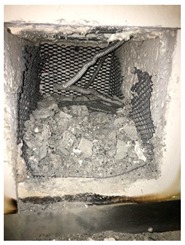	Exploded

**Table 12:** Available relationships for compressive strength and modulus of elasticity of unconfined normal and heavyweight concrete.

Reference	Compressive Strength and Modulus of Elasticity Equations
Carreira and Chu [64]	$E_{C}=0.0736w_{C}^{1.51}f_{C}^{\prime }{}^{0.3}$ $f_{CT}^{\prime }=f_{C}^{\prime }\beta x/\left(\beta -1+x^{\beta }\right)$ x=εCTεC; β=(fC′32.4)3+1.55
Yang et al. [63]	EC=A1fC′a(wCw0)b $A_{1}=8470;a=\frac{1}{3};\;b=1.17;\;w_{0}=2300\mathrm{kg}/m^{3}$
